# Evaluating Acute Testicular Pain Using Point-of-Care Hand-Held Doppler in the Emergency Department: A Prospective Pilot Study

**DOI:** 10.7759/cureus.17699

**Published:** 2021-09-03

**Authors:** Jagannath Hanumanthappa, Vamanjore A Naushad, Osama Mohammed, Ashok Kumar Ariboyina, Suresh Babu Chellapandian, Shoib Khan Mat Khan

**Affiliations:** 1 Emergency Medicine, Hamad Medical Corporation, Doha, QAT; 2 College of Medicine, Qatar University, Doha, QAT; 3 Internal Medicine, Weill Cornell, Doha, QAT; 4 General Internal Medicine, Hamad General Hospital /Hamad Medical Corporation, Doha, QAT; 5 Internal Medicine, Hamad Medical Corporation, Doha, QAT

**Keywords:** hand-held doppler, testicular pain, testicular torsion, emergency department, point-of-care, epididymitis, doppler ultrasound, radiologist doppler, epididymo-orchitis, orchitis

## Abstract

Introduction

Testicular pain is one of the common conditions in patients visiting the emergency department. The causes of acute testicular pain vary from non-urgent inflammatory conditions like epididymo-orchitis to testicular torsion which is a surgical emergency. Early diagnosis of testicular torsion with early initiation of appropriate surgical intervention helps in salvaging the testes. There is a need for a simple, rapid bedside diagnostic tool for the evaluation and triaging of subjects with acute testicular pain in the emergency department. The aim of this study is to determine whether hand-held Doppler (HHD) examination by the emergency department (ED) physician can safely rule out testicular torsion in a case of acute testicular pain.

Materials and Methods

A prospective pilot study was conducted in the emergency department of Alkhor Hospital, Hamad Medical Corporation, Qatar. The subjects between 18 to 50 years of age who presented to the ED with testicular pain were included. Subjects with recent trauma to the scrotum, or recent genitourinary surgery and those who had pain for more than 48 hours were excluded. Point-of-care HHD was done by a single ED physician who was blinded for the Doppler study results done by the radiologist. The results of the HHD performed by the ED physician and Doppler study performed by the radiologist were compared and analyzed.

Results

Forty-five patients were included in the study. The mean age was 28 years and the mean duration of pain was 20 hours. HHD ruled out testicular torsion in 44 subjects with a specificity of 97.8%. In one subject, HHD was reported as torsion testis which was ruled out by the radiologist. The radiologist Doppler ruled out torsion in all 45 subjects.

Conclusion

The diagnostic performance of HHD by the ED physicians was almost equal to that of radiologists in ruling out testicular torsion. HHD can be used as a first-line triaging tool by the ED physician to rule out torsion of testis in patients presenting with acute testicular pain. We conclude that patients with testicular pain with a negative HHD for torsion testis can be safely discharged from the emergency department.

## Introduction

Acute testicular pain is a common condition encountered by emergency physicians. Testicular torsion is a surgical emergency that needs to be considered in the differential diagnosis of acute testicular pain. Early diagnosis and appropriate intervention will help to salvage the organ. The estimated yearly incidence of testicular torsion was 5.9 per 100,000 between the age group one to 17 years and 1.3 per 100,000 in those older than 18 [1]. A large number of acute testicular pain cases are diagnosed as epididymitis, orchitis, epididymo-orchitis, and non-specific testicular pain [2-6]. These non-urgent inflammatory conditions can be conservatively managed and discharged from the emergency department (ED) without the need for Doppler examination by a radiologist with appropriate outpatient follow-up referrals. Doppler ultrasound of the testes is the investigation of choice to diagnose testicular torsion [7-9]. Routinely in most EDs, patients are referred to the radiology department for Doppler ultrasound; however, this is time-consuming and will delay the subsequent diagnosis and management of testicular pain. Subjecting all patients with acute testicular pain to a Doppler ultrasound to rule out torsion is not feasible as this will add to the burden of a single on-call radiologist and delay the disposition of these patients. Moreover, it will add to the bed scarcity of already overcrowded EDs. A simple, readily available, low-cost, and rapid point-of-care tool is required for the diagnosis by ED physicians. In this regard, the usefulness of hand-held Doppler (HHD) was evaluated as a point-of-care tool in the ED.

This study was conducted to determine the usefulness of HHD as a triaging tool for acute testicular pain by ED physicians, and the results were compared with those of Doppler examinations performed by a radiologist [10]. The objective was not to use HHD as the sole diagnostic test or to circumvent the need for Doppler ultrasound performed by radiologists but rather to determine whether HHD can play a role in the ED to alleviate the burden on the radiology department.

## Materials and methods

Study design and settings

A prospective pilot study was conducted in the emergency department of AlKhor Hospital, Hamad Medical Corporation, Qatar. Patients were enrolled from April 2018 to January 2020.

Study patients

The study included patients between 18 and 50 years of age who presented to the ED with testicular pain as a primary complaint. Patients who had pain for more than 48 hours, recent trauma to the testes, or recent genitourinary surgery were excluded from the study.

Study protocol

Patients presenting with acute testicular pain to the ED were triaged to the observation room by a triage nurse and assessed by an ED physician. The research protocol was initiated when the ED physician requested Doppler ultrasound by the radiologist to further evaluate acute testicular pain. The patients were screened by a research team member for the eligibility criteria. If eligible, they were enrolled in the study after obtaining informed consent. All patients were evaluated for a brief history, and a clinical examination was performed. History included duration and site of pain, fever, associated scrotal swelling, urinary complaints, and recent scrotal trauma or surgery. Clinical assessment included the area of tenderness, position of the testes, local erythema, and swelling. Laboratory investigations included urine analysis and complete blood cell counts.

HHD was performed by a single investigator who was an ED physician, and the findings were recorded. After the Doppler examination was performed by the radiologist and the results were documented, the HHD report was compared with that of the radiologist.

The investigators

The HHD study was performed by a single ED physician who was trained and certified in a point-of-care ultrasound course conducted by Hamad Medical Corporation. Since the HHD was performed and documented in the patient’s electronic file before the Doppler examination by the radiologist, the investigator was blinded to the radiology Doppler report. 

The device (HHD)

A BISTOS Hi-dop BT 200 Vascular Doppler (Gyeonggi-do, Korea) with an 8-MHz probe was used for the study.

Study methodology

HHD examination of the testes was performed with the patients in the supine position with the thighs abducted, and the testes were appropriately supported. The HHD examination was first performed on the unaffected side. The probe was initially placed over the upper pole of the testis and epididymis and then moved from medial to lateral to identify the testicular artery Doppler signals. In each of these regions, Doppler audio signal intensity was observed. The affected side was then examined similarly, and the audio signal intensity was compared with that of the normal side.

Inference

An increase in the Doppler audio signal intensity indicated increased blood flow to the region, suggesting inflammatory conditions such as epididymitis, orchitis, or epididymo-orchitis. On the contrary, diminished or absent Doppler audio signals indicated decreased or absent blood flow to the region, which is strongly diagnostic of testicular torsion.

The findings of HHD were documented in the patients’ electronic file to indicate possible testicular torsion or an inflammatory condition. The HHD results were compared with the radiologist's Doppler report, and the statistical significance was analyzed.

Ethical consideration

The study protocol was approved by the Institutional Review Board of Medical Research Center, Hamad Medical Corporation (Approval number-MRC 17055/17)

## Results

A total of 45 patients were included in the study. The mean age was 28 years (range 18-50 years), and the mean duration of pain was 20 hours. The majority of the patients had pain on the left side. Barring one patient, HHD performed by the ED physician was negative for testicular torsion. In contrast, the radiologist found all of the patients to be negative for testicular torsion (Table 1). HHD identified 14 and 31 patients with and without epididymo-orchitis, respectively compared with 18 and 27 respectively, by the radiologist (Tables 2, 3).

**Table 1:** HHD for testicular torsion HDD: Hand-held Doppler

	Positive	Negative
Handheld doppler	1	44
Radiologist doppler	0	45

**Table 2:** HHD for epididymo-orchitis HDD: Hand-held Doppler

	Positive	Negative
Handheld doppler	14	31
Radiologist doppler	18	27

**Table 3:** Specificity and sensitivity of HHD for epididymo-orchitis HDD: Hand-held Doppler

True positive	False positive
10	4
False negative	True negative
4	27

The mean duration of performing and reporting the results of HHD was 20 minutes, whereas it was 2 hours and 40 minutes for the Doppler examination performed by the radiologist.

Forty-four patients were discharged with medication. One patient was admitted for further workup because the radiology Doppler indicated a possible testicular tumor which was subsequently diagnosed as testicular malignancy.

By detecting the audible testicular artery signal at the level of the spermatic cord and upper level of the testis, HHD correctly excluded torsion in 44 cases with a specificity of 97.8%. Since testicular torsion was not encountered in the present study, other statistical variables could not be measured.

## Discussion

Our findings revealed that HHD, by detecting the audible testicular artery signal at the level of the spermatic cord and in the region just above the testis, excluded testicular torsion with a specificity of 97.8%. Although very few studies have been conducted on this subject, our results confirmed the results of previous studies. Shaikh et al., who studied the role of HHD in patients with testicular pain lasting less than 24 hours, had found that HHD had a sensitivity and specificity of 100% in diagnosing and excluding testicular torsion [3]. In another study by Mufti et al., HHD had a sensitivity of 100% and a specificity of 97% in diagnosing testicular torsion [4]. Both studies concluded that HHD is a reliable diagnostic tool for managing patients with acute testicular pain. The main difference between our study and the previous two studies is that none of our patients had a final diagnosis of testicular torsion. Hence, the sensitivity for diagnosing testicular torsion could not be calculated from our results.

One patient in the present study who was diagnosed with testicular torsion by HHD was found to be negative for torsion by the radiologist. On reviewing this patient, it was found that a thickened and edematous spermatic cord was likely the reason for the failure of HHD to detect the artery signal.

Point-of-care examination of the testes by HHD in the ED, for patients with acute testicular pain, has a wide range of clinical implications. Firstly, in routine practice, most of the patients with acute testicular pain are referred to the radiology department for Doppler ultrasound by ED physicians, despite relative certainty that the given condition is not testicular torsion on clinical examination. Referring all patients with testicular pain to the radiology department for Doppler ultrasound is not feasible and might delay the disposition of such patients from the ED. Hence, in patients with a positive testicular artery signal by HHD, unnecessary referral to the radiologist for Doppler ultrasonography can be avoided, which not only reduces the burden on the radiology department but is also cost-effective. Secondly, by ruling out testicular torsion, decisions about the management and disposition of patients with testicular pain can be made early. If HHD signals are clear, it most likely rules out testicular torsion, and these patients can be discharged from the ED with conservative management and outpatient clinic referrals [11-12]. On the contrary, if HHD signals are absent or diminished, testicular torsion is most likely the diagnosis. This will assist ED physicians in expediting further evaluation and intervention by a urologist [5,10,11-12]. Thirdly, our results revealed that the turnaround time for performing and reporting HHD was significantly less than that when a Doppler ultrasound is performed by a radiologist in the radiology department, which substantially reduced the length of stay in the ED. Finally, HHD will be useful in countries with poor healthcare resources, as it can be performed by general physicians with minimal training. Previous studies have shown that simple, readily available, and a non-invasive diagnostic tool such as a point-of-care HHD can act as supporting evidence to clinical examinations [3,4,13].

Significance of the study

The uniqueness of this study is that only limited studies have been conducted on the use of HHD for acute testicular pain, and no study has compared HHD with the Doppler ultrasound performed by the radiologist.

Limitations

The main limitation of the study was that none of our patients had testicular torsion; hence, the sensitivity of HHD in diagnosing torsion testes could not be ascertained. This could be due to the very low incidence of testicular torsion in this region. Another reason could be that the research was conducted in the adult ED, which excluded patients below 18 years of age. The incidence of testicular torsion is much higher in younger patients than in adults. Moreover, HHD was performed by a single researcher, and most of the cases were recruited during his work shifts. This further reduced the likelihood of recruiting cases of testicular torsion. Another limitation was the subjective nature of this investigative modality.

## Conclusions

HHD can be used as a first-line triaging tool by the ED physician to rule out testicular torsion in patients presenting with acute testicular pain. In addition, with minimal training required for the physicians, it can be a valuable tool to rule out testicular torsion in centers with poor healthcare resources. However, more extensive multi-center studies are needed to validate whether HHD can be used in day-to-day clinical practice to augment bedside clinical examination. Further studies with larger populations are also needed to ensure that an adequate number of testicular torsion cases are included to determine the usefulness of HHD.
